# Efficacy of NSAIDs and corticosteroids as premedication for post-endodontic pain management: A systematic review and meta-analysis

**DOI:** 10.4317/jced.63455

**Published:** 2025-12-30

**Authors:** Radia Mrini, Marcela Salamanca-Ramos, Priscilla Ledezma, Alejandro R. Pérez, José Aranguren, Javier Montecinos, Elías Utreras, Nicolas Pinto-Pardo

**Affiliations:** 1 Master in microscopic endodontics and apical surgery, Universidad Rey Juan Carlos, Alcorcón, Madrid, Spain; 2 Dentistry, Faculty of Life Sciences, Universidad Viña del Mar, Viña del Mar, Valparaiso, Chile; 3 Dentistry, Faculty of Dentistry and Rehabilitation Sciences, Universidad San Sebastián, Santiago, Chile; 4 Laboratory of Molecular and Cellular Mechanisms of Pain, Department of Biology, Faculty of Science, Universidad de Chile, Santiago, Chile

## Abstract

**Background:**

Post-endodontic pain remains a common clinical challenge. This systematic review and meta-analysis evaluated the preemptive efficacy of NSAIDs and corticosteroids as premedication for managing postoperative pain in patients with symptomatic irreversible pulpitis undergoing non-surgical root canal treatment. Objectives: To compare the preemptive efficacy of NSAIDs and corticosteroids as single-dose premedication in reducing postoperative pain and rescue medication use after endodontic treatment.

**Material and Methods:**

Following PRISMA 2020 guidelines, a systematic search was conducted in PubMed, Cochrane Library, and Embase up to April 2024. Eligibility criteria included randomized controlled trials comparing single-dose NSAIDs or corticosteroids with placebo. Primary outcomes were postoperative pain intensity measured by validated scales (VAS, NRS, HP-VAS) and need for rescue analgesics. Risk of bias was assessed with RoB2, and certainty of evidence with GRADE. Meta-analysis used random-effects models, with standardized mean differences (SMD) and 95% confidence intervals (CI). Protocol registered in PROSPERO (CRD42024499723).

**Results:**

Seven RCTs (n=820) published between 2009 and 2023 were included. Both corticosteroids (SMD = -1.28; 95 % confidence interval (CI): -1.96 to -0.61) and NSAIDs (SMD = -0.61; 95% CI: -1.11 to -0.10) significantly reduced postoperative pain versus placebo. NSAIDs provided rapid analgesia at 6 h, while corticosteroids achieved sustained analgesia from 12-48 h. Rescue analgesic use decreased substantially in both active groups compared with placebo (NSAIDs 4.6%, corticosteroids 5.5%, placebo 34.4%). High heterogeneity (I² = 90%) was partly explained by drug class, dose, and administration route. Sensitivity analysis excluding imputed data confirmed robustness of results.

**Conclusions:**

Both NSAIDs and corticosteroids are effective preemptive agents for managing post-endodontic pain in symptomatic irreversible pulpitis. NSAIDs should be preferred for rapid early relief, while corticosteroids provide extended analgesia. Findings highlight their opioid-sparing potential and support their inclusion in evidence-based endodontic pain management protocols. Further multicenter RCTs are warranted to refine patient-tailored regimens.

## Introduction

Orofacial pain, prevalent in 5% to 57% of individuals across various regions (1), significantly impacts psychological, social, and economic well-being, with dental pain affecting 7% to 32% of the global population (2). Dental pain primarily arises from periapical or pulp conditions, often requiring endodontic therapy or extraction (3). Around 81% of these cases necessitate endodontic treatment (4), with the primary goal being the rapid relief of pain, particularly in cases of symptomatic irreversible pulpitis (5). Symptoms include persistent thermal discomfort, spontaneous pain, and referred pain, sometimes with no overt clinical signs despite underlying inflammation (6). Irreversible pulpitis is an inflammatory condition of the dental pulp, primarily triggered by bacterial infections (7). The immune response involves pro-inflammatory cytokines (such as IL-8 and TNF-) (8) and gene expression of IL-6 and MMP-9, driving inflammation and tissue degradation (9). Immune cell interactions, such as those involving calcitonin receptors, also contribute to pulp inflammation and pain (7 , 8). Non-surgical root canal therapy is the recommended treatment for irreversible pulpitis in mature permanent teeth, guided by clinical indicators and patient-reported experiences (10). Postoperative pain, affecting 3-58% of patients, requiring effective analgesic and anti-inflammatory strategies (2 , 11 , 12). Preemptive pain management is crucial, as preoperative medication can reduce postoperative pain by preventing central and peripheral sensitization (13 , 14). Effective premedication includes analgesia plans and patient education to minimize acute discomfort and prevent chronic pain (15). Analgesics are classified as opioids and non-opioids. While opioids like morphine or tramadol manage moderate to severe pain, they have significant side effects and dependency risks (16). Non-opioids, such as corticosteroids and NSAIDs, are essential due to their anti-inflammatory properties (17 , 18). Corticosteroids (dexamethasone or prednisolone) inhibit phospholipase A2, reducing inflammation (19). NSAIDs (ibuprofen or naproxen), act on cyclooxygenase enzymes, decreasing prostaglandin production and inflammation (20). However, NSAIDs may cause gastrointestinal issues, necessitating cautious prescribing (21). Continuous medication use raises risks of gastrointestinal bleeding and renal impairment, emphasizing the need for optimized medication strategies (22). A systematic review and meta-analysis are needed to develop clear clinical guidelines. Optimizing premedication with corticosteroids and NSAIDs may significantly enhance patient outcomes and satisfaction following endodontic treatments. Population, Intervention, Comparator, Outcomes (PICO): Adults with symptomatic irreversible pulpitis undergoing nonsurgical root canal treatment; single-dose premedication with NSAIDs or corticosteroids; comparator groups were placebo or the alternative drug class; primary outcomes were postoperative pain intensity at 6, 12, 24, and 48 h, and the use of rescue analgesics. Only randomized controlled trials were eligible. This review adds novelty by integrating a direct head-to-head quantitative comparison between corticosteroids and NSAIDs, accompanied by time-dependent (6-48 h) efficacy analysis and synthesis of rescue-medication use-an approach not addressed in previous meta-analyses.

## Material and Methods

This systematic review and meta-analysis followed the PRISMA 2020 guidelines. The protocol was prospectively registered in PROSPERO (CRD42024499723). Eligibility criteria were defined according to the PICO framework described in the Introduction. This review included only randomized controlled trials (RCTs) enrolling adults diagnosed with symptomatic irreversible pulpitis (SIP), with or without symptomatic apical periodontitis, requiring nonsurgical root canal treatment under local anesthesia. Eligible studies met the following PICO-based criteria: Population (P): Adult patients (18 years) with a clinical and radiographic diagnosis of symptomatic irreversible pulpitis in permanent teeth. Intervention (I): Single-dose premedication with nonsteroidal anti-inflammatory drugs (NSAIDs) or corticosteroids administered orally, intramuscularly, or submucosally prior to endodontic treatment. Comparator (C): Placebo or an alternative drug class (NSAID vs corticosteroid). Outcomes (O): Primary outcome-postoperative pain intensity assessed at 6, 12, 24, and 48 h after treatment using validated scales (VAS, NRS, HP-VAS). Secondary outcome-the need for rescue analgesic medication. Only studies that clearly reported baseline and postoperative pain scores, drug dosage and administration route, and follow-up periods were included. Exclusion criteria were: (1) Non-randomized studies, quasi-experimental designs, case reports, or reviews; (2) Trials involving patients with systemic diseases, pregnant or lactating women, or those under chronic anti-inflammatory, analgesic, or corticosteroid therapy; (3) Studies without quantitative pain data or lacking mean and standard deviation values for each time point; (4) Trials where premedication was part of combination therapy with antibiotics or other analgesics; (5) Non-English publications or inaccessible full-text articles. Search strategy: A comprehensive search was conducted in PubMed, Cochrane Library, and Embase up to April 2024, combining controlled vocabulary (MeSH/Emtree) and free-text terms for "NSAIDs," "corticosteroids," "pulpitis," and "endodontic pain." Detailed strategies are provided in the (Supplement 1) http://www.medicinaoral.com/medoralfree01/aop/jced_63455_s01.pdf No language or publication status restrictions were applied. A list of excluded full-text studies with reasons is provided in (Supplement 2) http://www.medicinaoral.com/medoralfree01/aop/jced_63455_s02.pdf Certainty of evidence for each main outcome and comparison is summarized using the GRADE (Supplement 3) http://www.medicinaoral.com/medoralfree01/aop/jced_63455_s03.pdf For the head-to-head comparison (corticosteroids vs NSAIDs), we pooled only direct randomized evidence; no network meta-analysis was performed. Random-effects models were used throughout. Study selection: Three reviewers independently screened titles and abstracts, followed by full-text assessment. Disagreements were resolved by discussion and arbitration by a senior reviewer. Duplicates were removed with Mendeley software. The PRISMA 2020 flow diagram summarizes the process. Data extraction: Using a standardized Excel form, three reviewers independently extracted study characteristics (author, year, country, sample size, demographics, intervention, comparator, outcomes, and follow-up). The primary outcomes were postoperative pain intensity at 6, 12, 24, and 48 hours (measured by VAS, NRS, or HP-VAS) and need for rescue medication. Secondary outcomes included pain trajectories over time. Risk of bias and certainty: The Cochrane RoB2 tool was applied to assess risk of bias across domains: randomization, deviations from interventions, missing data, outcome measurement, and reporting. Certainty of evidence was graded with the GRADE approach, considering risk of bias, inconsistency, indirectness, imprecision, and publication bias. Statistical analysis: Meta-analyses were conducted using RevMan 5.4. Outcomes were pooled as standardized mean differences (SMD) with 95% confidence intervals (CI). Given substantial heterogeneity (I² = 90%), a random-effects model was applied. Subgroup analyses explored drug class (NSAIDs vs. corticosteroids), specific agent, dose, and administration route. To address concerns regarding imputed values from exponential decay modeling, a sensitivity analysis excluding imputed points was performed (Supplement 4) http://www.medicinaoral.com/medoralfree01/aop/jced_63455_s04.pdf Publication bias was assessed with funnel plots and Egger's test (p = 0.12).

## Results

**Figure 1:**
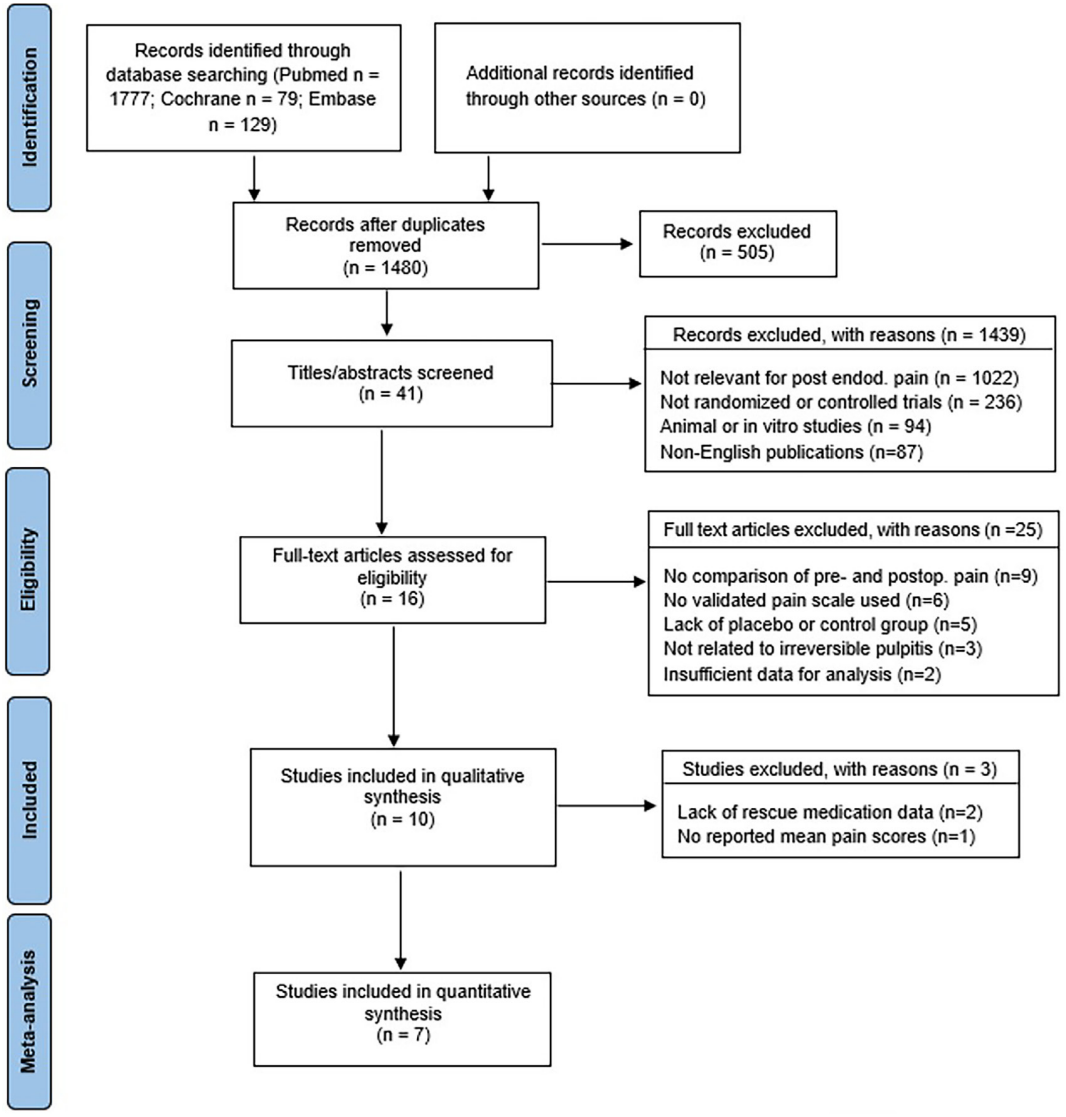
PRISMA 2020 flow diagram illustrating the study selection process.

Study selection: The initial search retrieved 1,327 records. After removal of duplicates and screening, 24 full-text articles were assessed. Finally, 7 randomized controlled trials (n = 820) published between 2009 and 2023 met the inclusion criteria. The PRISMA 2020 flow diagram summarizes the process (Fig. 1).

1

Flowchart of identification, screening, eligibility, and inclusion of randomized controlled trials evaluating single-dose premedication (NSAIDs or corticosteroids) for post-endodontic pain in symptomatic irreversible pulpitis. Reasons for exclusion are shown at each stage according to PRISMA 2020. Seven RCTs were included in the quantitative synthesis. Study characteristics: Included RCTs investigated different NSAIDs (ibuprofen, ketorolac, diclofenac) and corticosteroids (dexamethasone, prednisolone, betamethasone) administered as a single oral or intramuscular premedication before non-surgical root canal treatment in patients with symptomatic irreversible pulpitis. Placebo served as the comparator in all trials. Pain was measured using validated scales (VAS, NRS, HP-VAS) at 6, 12, 24, and 48 hours postoperatively. Rescue analgesic intake was recorded in most studies. Table 1 summarizes patient numbers, pre-medication used, and outcome verification methods.

Table 1

Risk of Bias Assessment: The Revised Cochrane Risk of Bias Tool (RoB2) assessed study quality (Table 2).

Table 2

**Figure 2:**
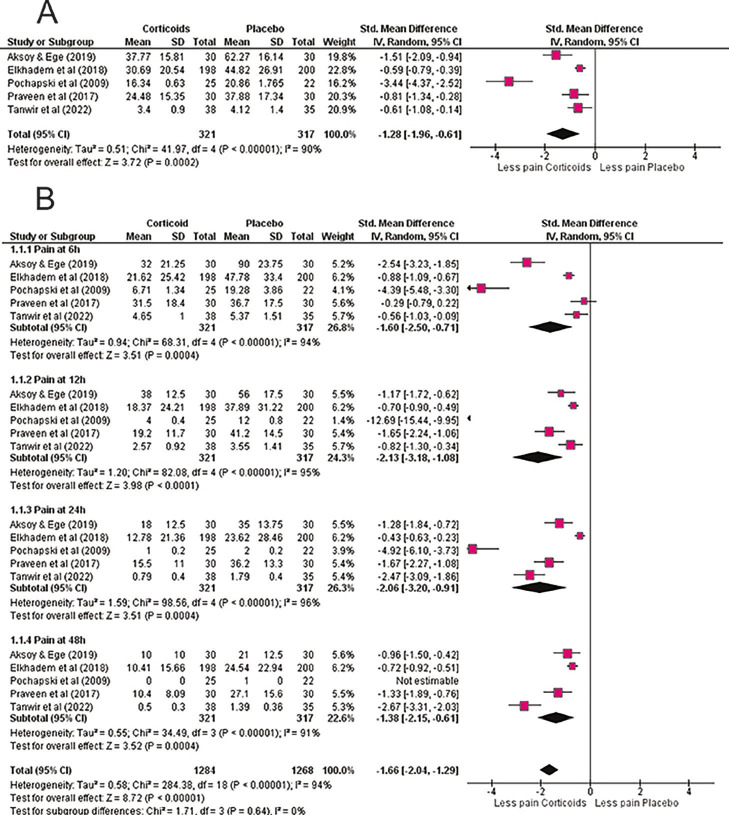
Corticosteroid premedication versus placebo. (A) Overall random-effects meta-analysis comparing corticosteroids with placebo for postoperative pain after endodontic treatment. Effect sizes are standardized mean differences (SMD) with 95% confidence intervals (negative values favor corticosteroids). Across the included trials (corticosteroids n=321; placebo n=317), corticosteroid premedication significantly reduced pain (overall SMD = –1.28). Substantial heterogeneity likely reflects variation in drug type, dose, and route. (B) Time-course analysis (6, 12, 24, 48 h). Corticosteroids show rapid benefit that is most evident at 12–24 h and remains present at 48 h, consistent with their sustained anti-inflammatory action.

Most studies were low risk of bias, though some had "some concerns" in randomization and missing outcome data. Summary of Included Studies: Primary outcomes measured were pain relief efficacy and duration of postoperative pain control. These studies provide insights into premedication strategies for managing postoperative pain during endodontic procedures. Meta-analysis Corticoids vs Placebo: Meta-analysis of seven RCTs demonstrated that premedication with corticosteroids significantly reduced postoperative endodontic pain compared with placebo (SMD = -1.28; 95% CI: -1.96 to -0.61; p = 0.0002), despite high heterogeneity (I² = 90%) (Fig. 2A).

2

**Figure 3:**
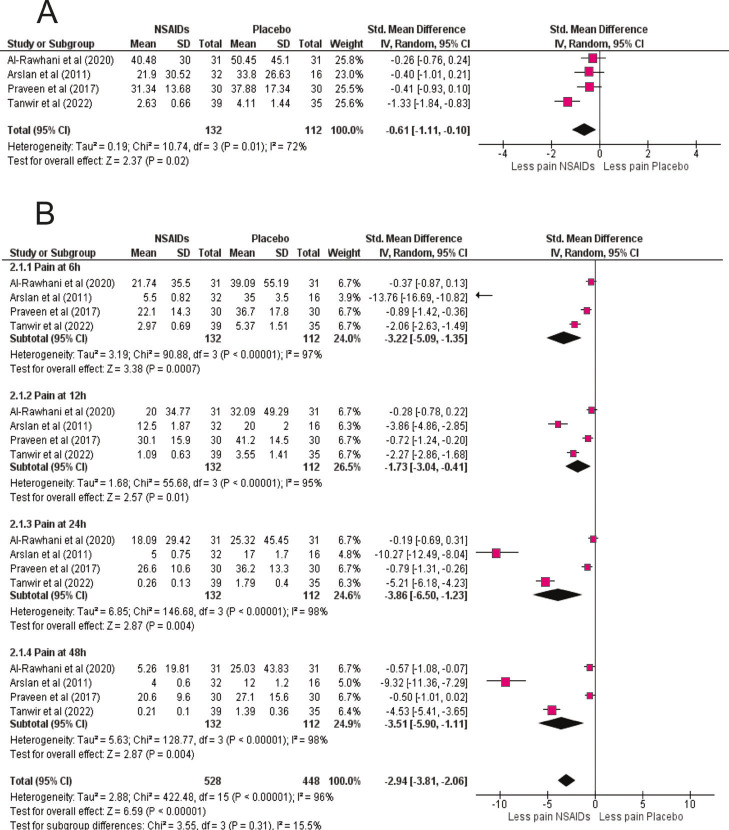
NSAID premedication versus placebo. (A) Overall random-effects meta-analysis comparing NSAIDs with placebo for postoperative pain. SMDs (95% CI) are shown; negative values favor NSAIDs. Pooled results (NSAIDs n=132; placebo n=112) demonstrate a significant reduction in pain (overall SMD ≈ –0.61), with moderate–high heterogeneity attributable to differences in drug and dosing regimens. B) Time-course analysis (6, 12, 24, 48 h). NSAIDs provide the greatest early analgesic effect at 6 h, with clinically meaningful reductions persisting through 24–48 h, consistent with their rapid onset via COX inhibition.

Prednisolone (20-40 mg), dexamethasone (4-8 mg, oral or intramuscular), and methylprednisolone were tested, all showing consistent analgesic benefit. Time-course analysis confirmed efficacy at all intervals. At 6 h, corticosteroids reduced pain substantially (SMD = -1.60; 95% CI: -2.50 to -0.71; p = 0.0004), with effects maintained at 12 h (SMD = -2.13; 95% CI: -3.18 to -1.08; p &lt; 0.0001), 24 h (SMD = -2.06; 95% CI: -3.20 to -0.91; p = 0.0004), and 48 h (SMD = -1.38; 95% CI: -2.15 to -0.61; p = 0.001) (Fig. 2B). These findings highlight both rapid and sustained corticosteroid analgesia, supporting their role as an effective preemptive option in endodontic pain management. NSAIDs vs Placebo: NSAID premedication also significantly reduced postoperative pain compared with placebo (SMD = -0.61; 95% CI: -1.11 to -0.10; p = 0.02; I² = 72%) (Fig.3A).

3

**Figure 4:**
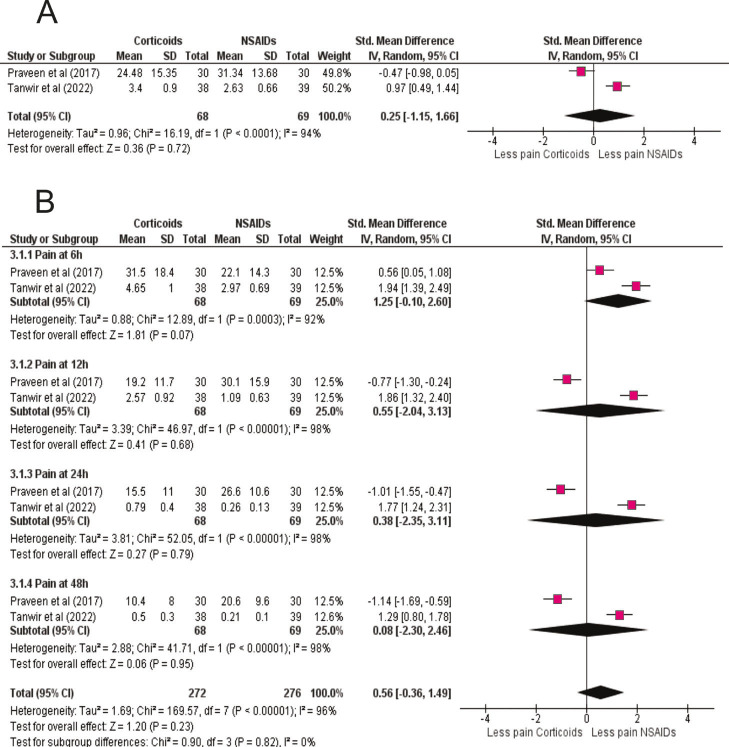
Corticosteroids versus NSAIDs (head-to-head comparison). (A) Overall random-effects meta-analysis directly comparing corticosteroids and NSAIDs for postoperative pain reduction. SMDs (95% CI) are shown; negative values favor corticosteroids. No statistically significant difference was observed (corticosteroids n=68; NSAIDs n=69), indicating broadly comparable analgesic efficacy. (B) Time-course comparison (6, 12, 24, 48 h). NSAIDs tend to provide faster relief at 6 h, whereas corticosteroids maintain analgesia into the first two postoperative days, illustrating complementary onset–duration profiles relevant to individualized premedication.

Trials tested ibuprofen (200 mg), diclofenac potassium (50 mg), and tenoxicam (20 mg), all demonstrating superiority over placebo. Time-course analysis revealed the strongest effect at 6 h (SMD = -3.22; 95% CI: -5.09 to -1.35; p = 0.0007), with continued benefit at 12 h (SMD = -1.73; 95% CI: -3.04 to -0.41; p = 0.01), 24 h (SMD = -3.86; 95% CI: -6.50 to -1.23; p = 0.004), and 48 h (SMD = -3.51; 95% CI: -5.90 to -1.11; p = 0.004) (Fig 3B). These results confirm NSAIDs' rapid onset of analgesia, consistent with their cyclooxygenase-inhibiting mechanism. Corticoids vs NSAIDs: Direct comparison between corticosteroids and NSAIDs revealed no statistically significant difference (SMD = 0.25; 95% CI: -1.15 to 1.66; p = 0.72; I² = 94%) (Fig. 4A).

4

Some trials slightly favored NSAIDs (Praveen et al.), while others favored corticosteroids (Tanwir et al.), reflecting heterogeneity in drug, dose, and administration. Time-course comparisons showed NSAIDs had a trend toward stronger pain reduction at 6 h (SMD = -1.25; 95% CI: -2.60 to 0.10; p = 0.07), while corticosteroids provided more sustained analgesia at later intervals, although differences were not statistically significant (Fig. 4B). These findings suggest both drug classes are effective, with NSAIDs better suited for early relief and corticosteroids for prolonged control. Use of Rescue Medication after Endodontic Treatment: Rescue medication use was consistently lower in premedicated arms than in placebo. Across trials, placebo groups showed the highest proportion of patients requiring additional analgesics (typically 30%), whereas both NSAID- and corticosteroid-premedicated groups were below 10%. These patterns were observed irrespective of drug, dose, and route, and remained robust in sensitivity analyses (Supplement 5) (http://www.medicinaoral.com/medoralfree01/aop/jced_63455_s05. Modeling of missing intermediate time points.). ANOVA and Tukey post-hoc analyses confirmed significant differences versus placebo (p &lt; 0.05), but no difference between active treatments (p = 0.99). Individual studies echoed these results: Elkhadem et al. reported 48% of placebo patients versus 22% with prednisolone; Aksoy &amp; Ege reported 56.7% with placebo versus 13.3% with dexamethasone; Al Rawhani et al. observed dose-dependent reductions with ketorolac (20% placebo vs 7.5% at 10 mg and 5% at 20 mg). Across all studies, both NSAIDs and corticosteroids substantially decreased reliance on supplementary analgesia (Table 3).

Table 3

Pain Perception Over Time: Two-way ANOVA demonstrated that time accounted for most variation in pain scores (78.1%, p &lt; 0.0001), with a significant effect of treatment type (p = 0.0019) but no significant interaction (p = 0.47). ídák's test showed NSAIDs significantly reduced pain versus placebo at 6 h (p = 0.0106). Corticosteroids also lowered pain scores at this interval but without statistical significance (p = 0.12). At 12, 24, and 48 h, pain scores declined similarly across groups, with no significant differences between active treatments. This pattern reinforces NSAIDs' role in early postoperative relief and corticosteroids' contribution to longer-term control (Supplement 5) http://www.medicinaoral.com/medoralfree01/aop/jced_63455_s05.pdf

## Discussion

This systematic review and meta-analysis evaluated the preemptive use of corticosteroids and NSAIDs for managing postoperative endodontic pain in symptomatic irreversible pulpitis. Unlike prior reviews that examined each drug class separately, this study provides the first integrated quantitative comparison encompassing temporal efficacy (6-48 h) and rescue-medication outcomes. This unified framework clarifies the time-dependent analgesic profiles of both drug classes and supports evidence-based, opioid-sparing strategies. The findings confirm that both corticosteroids and NSAIDs significantly reduce pain intensity and the need for additional analgesics compared with placebo, underscoring their clinical relevance in acute dental pain management (23 - 25). This work advances previous evidence by offering a head-to-head synthesis that enhances clinical decision-making through a comprehensive appraisal of onset, duration, and overall analgesic effectiveness. The finding that NSAIDs produced rapid pain relief within 6 h, while corticosteroids exerted more sustained effects up to 48 h, reflects the pharmacodynamic differences between these drug classes. NSAIDs inhibit cyclooxygenase enzymes, thereby suppressing prostaglandin synthesis and reducing peripheral nociceptor sensitization (26). This mechanism explains their superior early performance, particularly with agents such as ibuprofen and diclofenac, which have short plasma half-lives but rapid onset of action (27). In contrast, corticosteroids act through glucocorticoid receptor activation, leading to downregulation of pro-inflammatory cytokines such as IL-6 and TNF-, and upregulation of IL-10 (28). These genomic effects suppress NF-B pathways, reduce vascular permeability, and dampen neurogenic inflammation, resulting in delayed but prolonged analgesia (29 , 30). The distinct temporal patterns observed in our analysis therefore have a clear biological rationale and support the potential role of multimodal regimens that combine both drug classes to maximize early and sustained pain relief (31). This mechanistic complementarity may explain why both drug classes ultimately converge in analgesic efficacy but differ in onset and duration, informing clinical decisions for individualized premedication strategies. Our findings align with several previous systematic reviews and randomized controlled trials. Shamszadeh et al. reported consistent reductions in postoperative endodontic pain with corticosteroid premedication, particularly at 12 and 24 hours (32). Similarly, Jose et al. demonstrated in a systematic review that both NSAIDs and corticosteroids administered orally are effective in reducing post-endodontic pain, with comparable efficacy across most time intervals (25). Hegde et al. confirmed the benefit of preoperative corticosteroids in single-visit root canal treatment, reinforcing their role in difficult-to-manage pulpitis cases (24). However, individual trials have shown conflicting results. Zanjir et al. observed that NSAIDs maintained superior pain reduction at later intervals (33), whereas Konagala et al. found no significant difference beyond 12 hours (34). Tanwir et al. reported that both piroxicam and prednisolone were effective without major differences (35). These inconsistencies highlight the variability in drug type, dosage, administration routes, and study methodology, which were reflected in the high heterogeneity (I² &gt; 70%) of our pooled results. The clinical implications of these findings are significant. The 2024 American Dental Association (ADA) guideline strongly recommends NSAIDs, alone or in combination with acetaminophen, as first-line therapy for acute dental pain and discourages opioid prescribing (23). Our results confirm the rapid analgesic benefits of NSAIDs, particularly important in acute cases where immediate relief is needed. However, the sustained analgesic profile of corticosteroids suggests an additional role in situations where prolonged control is desirable or where NSAIDs are contraindicated due to gastrointestinal or renal risks (36). These insights support individualized prescribing decisions in endodontic practice and encourage consideration of multimodal strategies. Rescue medication use provided further confirmation of the effectiveness of both drug classes. Placebo groups consistently showed the highest rates of supplemental analgesic intake, often exceeding 30%, whereas premedication with either NSAIDs or corticosteroids reduced this to below 10% (24 , 35 , 37). For example, Elkhadem et al. demonstrated that preoperative prednisolone halved the number of patients requiring additional analgesics after root canal treatment (37). These findings illustrate not only improved patient comfort but also an important opioid-sparing effect. In the context of the ongoing opioid crisis, the capacity of NSAIDs and corticosteroids to reduce the need for supplementary pain medication strengthens the case for their routine use in endodontic protocols (23). It is also important to recognize that pain perception is influenced not only by pharmacological interventions but also by psychosocial factors. Placebo responses in dental pain can reach up to 30% and are mediated by endogenous opioid activity and anticipatory mechanisms (38 , 39). Anxiety, prior dental experiences, and patient expectations strongly modulate perceived pain intensity (40). These considerations emphasize the value of combining pharmacological approaches with effective communication and reassurance to optimize outcomes, particularly in anxious patients. Although this review adhered to PRISMA standards and applied RoB2 and GRADE, certainty is tempered by small sample sizes, single-center designs, and heterogeneity in drug type, dose, route, and outcome measurement. According to the GRADE assessment (Supplement 3) (http://www.medicinaoral.com/medoralfree01/aop/jced_63455_s03), the certainty of evidence ranged from moderate to low, mainly due to heterogeneity and sample size limitations, yet the consistency of effect direction supports clinical applicability. These limitations underscore the need for larger, multicenter randomized controlled trials with standardized protocols to strengthen the evidence base.

## Conclusions

According to the findings of the present systematic review and meta-analysis, both NSAIDs and corticosteroids are effective preemptive medications for reducing postoperative pain in symptomatic irreversible pulpitis. NSAIDs provide rapid early relief, while corticosteroids sustain analgesia into the first two postoperative days. Both significantly reduce the need for rescue analgesics, making them valuable tools in opioid-sparing pain management strategies. Clinicians should tailor drug selection based on patient needs, contraindications, and desired timing of pain control. By integrating pharmacological evidence with guideline recommendations and psychosocial strategies, endodontic practice can further optimize patient-centered pain management. Clinicians should consider NSAID premedication for immediate pain control and corticosteroids when extended postoperative relief is desirable, particularly in cases where NSAIDs are contraindicated. Future multicenter RCTs should aim to define optimal dosing and combined regimens to balance efficacy and safety in endodontic pain management. These findings reinforce the evidence base for incorporating preemptive anti-inflammatory strategies into routine endodontic protocols.

## Figures and Tables

**Table 1:** Table Characteristics of included randomized controlled trials.

Author	Year	Country	Type of study	Operator	Sample size	Sex	Age	Tooth	Premedication	Control	Follow-up period	Method of Outcome Verification
Pochapski et al.	2009	Brazil	Randomized Clinical Trial	Not specified	50 patients	26 males, 24 females	18-67 years	All	Dexamethasone (4 mg)	Placebo	4, 12, 24, 48h post- treatment	NRS (0-100mm)
Arslan et al.	2011	Turkey	Randomized Double Blinded Clinical Trial	Endodontic resident	48 patients	32 females, 16 males	18-52 years	All	Tenoxicam (20 mg), Ibuprofen (200 mg)	Placebo	6, 12, 24, 48, 72hours post- treatment	VAS (0-100 mm)
Praveen et al.	2017	India	Single CentreRandomizedControlled Trial	Endodonticresidents	42 patients	Ketorolacgroup: 8 males,6 females;Prednisolonegroup: 9 males,6 females;Placebo group:7 males, 6females	18-51 years	All	Ketorolac (20mg),Prednisolone(30 mg)	Placebo	6, 12, 24, 48h post-treatment	VAS (0-100mm)
Elkhadem et al.	2018	Egypt	Single CentreRandomized Controlled Trial	Endodontic residents	400 patients	Prednisolonegroup: 78males, 122females;Control group:63 males, 137 females	18-35 years	Mandibular molars	Prednisolone (40 mg)	Placebo	6, 12, 24 h post-treatment	VAS (0-100 mm)
Aksoy & Ege.	2019	Turkey	RandomizedControlledClinical Trial	Endodonticresidents	90 patients	Control: 13males, 17females;Tramadol: 18males, 12females;Dexamethasone14 males, 16females	18-65 years	Mandibularmolars	Tramadol (100mg/2 ml),Dexamethasone(8 mg/2 ml)	Saline (2 ml)	6, 12, 24, 48, 72h post-treatment	HP-VAS (0-170 mm)
Al-Rawhani et al.	2020	Egypt	RandomizedPlacebo-ControlledDouble-BlindTrial	Not specified	70 patients	55 females, 15males	>18 years	Mandibularmolars	DiclofenacPotassium (50mg)	Placebo (n=34)	6, 12, 24, 48h post-treatment	HP-VAS (0-170 mm)
Tanwir et al.	2022	Pakistan	Randomized Clinical Trial	Dental professionals	120 patients	71 females, 41 males	10-40 years	ingle rooted tooth	Piroxicam (20 mg), Prednisolone (20 mg)	No medication	96 h post- treatment	VAS (0-10 mm)

Summary of seven RCTs (n = 820) comparing single-dose premedication with NSAIDs or corticosteroids against placebo for post-endodontic pain. Details include author, year, country, sample size, drug regimen, comparator, pain-assessment method (VAS/NRS/HP-VAS), and follow-up intervals.

**Table 2:** Table Risk of bias assessment of included studies.

Author	Bias arising from the randomization process	Bias due to deviations from interventions	Bias due to missing outcome data	Bias in measurement of the outcome	Bias in selection of the reported result	Overall Bias
Pochapski et al.	-	-	-	-	?	?
Arslan et al.	-	-	-	-	-	-
Praveen R et al.	-	-	-	-	?	?
Elkhadem A. et al.	-	-	-	-	-	-
Aksoy & Ege.	-	-	-	-	?	?
Al-Rawhani et al.	-	-	-	-	-	-
Tanwir A. et al.	-	-	-	-	-	?
	-	Low risk				
		High risk				
	?	Some concerns				

Risk of bias assessment of included trials using the Cochrane RoB2 tool. Symbols indicate: ‘–’ low risk, ‘?’ some concerns, ‘+’ high risk. Most studies were low risk, though some showed concerns in randomization or missing data

**Table 3:** Table Rescue medication usage across included trials.

Study	Group	Number of patients	Rescue medication	Dosage	Patients taking rescue medication	Percentage	Timing of medication usage
Elkhadem et al.	Placebo	200	Analgesic	50 mg	96	48%	30 minutes before procedure
Elkhadem et al.	Prednisolone	200	Analgesic	50 mg	44	22%	30 minutes before procedure
Aksoy & Ege.	Placebo	30	Ibuprofen	400 mg	17	56.7%	Submucosal injection before procedure
Aksoy & Ege.	Dexamethasone	30	Ibuprofen	400 mg	4	13.3%	Submucosal injection before procedure
Al Rawhani et al.	Placebo	40	Paracetamol	500 mg	8	20%	1 hour before procedure
Al Rawhani et al.	Ketorolac (10 mg)	40	Paracetamol	500 mg	3	7.5%	1 hour before procedure
Al Rawhani et al.	Ketorolac (20 mg)	40	Paracetamol	500 mg	2	5%	1 hour before procedure
Tanwir et al.	Placebo	40	Ibuprofen	200 mg	5	12.5%	30 minutes before procedure
Tanwir et al.	Piroxicam	40	None	-	0	0%	30 minutes before procedure
Tanwir et al.	Prednisolone	40	None	-	0	0%	30 minutes before procedure

Summary of supplementary analgesic use across treatment groups. Data include number of patients requiring additional medication, dosage, and percentage by group. Placebo arms consistently showed higher rescue medication use, confirming superior preventive efficacy of both NSAIDs and corticosteroids.

## Data Availability

Data available on request from the authors.
